# Association between cerebrospinal fluid volume and frailty in community-dwelling older adults: a cross-sectional study

**DOI:** 10.1186/s12987-026-00761-1

**Published:** 2026-01-24

**Authors:** Kazuhiro Yoshiura, Yosuke Hidaka, Takashi Suehiro, Naoto Kajitani, Asuka Koyama, Yusuke Miyagawa, Naoko Tsunoda, Tomohisa Ishikawa, Ryuji Fukuhara, Mamoru Hashimoto, Manabu Ikeda, Megumi Shimodozono, Kazunari Ishii, Minoru Takebayashi

**Affiliations:** 1 Department of Frailty Research, Center for Gerontology and Social Science, Research Institute, National Center for Geriatrics and Gerontology, 7-430 Morioka-cho, Obu, Aichi 474-8511 Japan; 2 Department of Psychiatry, Osaka University Graduate School of Medicine, Osaka, Japan; 3 Department of Psychiatry and Neuroscience, Center for Metabolic Regulation of Healthy Aging, Faculty of Life Sciences, Kumamoto University, Kumamoto, Japan; 4 Faculty of Social Welfare, Kumamoto Gakuen University, Kumamoto, Japan; 5 Department of Psychiatry, Kumamoto Seimei Hospital, Kumamoto, Japan; 6 Department of Neuropsychiatry, Faculty of Life Sciences, Kumamoto University, Kumamoto, Japan; 7 Department of Geriatric Psychiatry, Mitsugumachi Clinic, Kumamoto, Japan; 8 Department of Psychiatry, Arao Kokoronosato Hospital, Arao, Japan; 9 Department of Psychiatry, Kagoshima University Graduate School of Medical and Dental Sciences, Kagoshima, Japan; 10 Department of Neuropsychiatry, Kindai University Faculty of Medicine, Sakai, Japan; 11 Department of Rehabilitation and Physical Medicine, Kagoshima University Graduate School of Medical and Dental Sciences, Kagoshima, Japan; 12 Department of Radiology, Kobe University Graduate School of Medicine, Kobe, Japan

**Keywords:** Frailty, Disproportionately enlarged subarachnoid-space hydrocephalus, Cerebrospinal fluid dynamics, Idiopathic normal-pressure hydrocephalus, Magnetic resonance imaging

## Abstract

**Background:**

Impaired cerebrospinal fluid (CSF) dynamics may affect brain health in older adults and contribute to age-related changes in brain structure. Disproportionately enlarged subarachnoid-space hydrocephalus (DESH) is a neuroimaging finding associated with impaired CSF dynamics. However, the association between frailty—a condition characterised by increased vulnerability in late life—and DESH-related CSF space volumes remains poorly understood. Therefore, in this study, we aimed to investigate this association.

**Methods:**

This cross-sectional study was conducted using data from 1,395 community-dwelling Japanese adults aged ≥ 65 years without dementia. Frailty was assessed using the Japanese version of the Fried criteria, comprising slowness, weakness, low activity, shrinking, and exhaustion. Volumes of regions of interest (ROIs) were measured by magnetic resonance imaging, and DESH-related regions (ventricular system [VS], Sylvian fissures [SF], and the subarachnoid space at the high convexity and midline [SHM]) were quantified using voxel-based morphometry.

**Results:**

Ordinal logistic regression analysis was conducted with frailty status (robust [reference], prefrail, or frail) as the dependent variable and each ROI volume as independent variables. The results showed positive associations of VS volume (odds ratio [OR] 1.21; 95% confidence interval [CI] 1.06–1.37) and SF volume (OR 1.81, 95% CI 1.09–3.02) with frailty, whereas SHM volume was negatively associated (OR 0.84, 95% CI 0.72–0.97). Logistic regression analyses of each frailty component and ROIs showed that slowness (slow gait speed) was consistently associated with all three DESH-related regions (false-discovery rate-adjusted q < 0.05).

**Conclusions:**

DESH-related CSF space volumes, reflecting impaired CSF dynamics, were significantly associated with frailty in community-dwelling older adults. These findings highlight the potential role of CSF dynamics as a neural mechanism underlying frailty and suggest a novel target for preventive strategies.

**Supplementary Information:**

The online version contains supplementary material available at 10.1186/s12987-026-00761-1.

## Background

Global population ageing has become a significant issue, with frailty representing an important concept in promoting healthy longevity among older adults [1]. Frailty is a pathological condition involving increased vulnerability to external stressors associated with ageing and is known to elevate the risk of various adverse outcomes, including dementia and mortality [2]. The mechanisms underlying frailty are multifactorial, and from a neurological perspective, numerous studies have shown associations between frailty and changes in the brain parenchyma [3].

In contrast, recent discoveries of mechanisms such as the glymphatic system [4], which is responsible for clearing metabolic waste from the brain, have revealed that cerebrospinal fluid (CSF) dynamics play a crucial role in brain ageing [5]. One disease associated with disturbances in CSF dynamics is idiopathic normal pressure hydrocephalus (iNPH; Hakim’s disease [6]), characterised by the clinical triad of cognitive impairment, gait disturbance, and urinary incontinence [7]. Symptoms of iNPH improve with CSF shunting, indicating that age-related disturbances in CSF dynamics are involved in its pathophysiology [8]. Disproportionately enlarged subarachnoid-space hydrocephalus (DESH) is a characteristic imaging finding in iNPH—defined by enlargement of the ventricular system (VS), widening of the Sylvian fissures (SF), and narrowing of the subarachnoid space at the high convexity and midline (SHM)—and can be regarded as an indirect imaging marker of CSF dynamic disturbances [9]. Among community-dwelling older adults, 15.8% have been reported to exhibit DESH-related findings [10]. As most of these individuals do not present with overt neurological symptoms, the clinical relevance of such findings has garnered increasing attention [10, 11].

Vallet et al. [12] reported a significant association between the brain elastic modulus derived from the CSF infusion test and frailty index, suggesting that the biomechanical response of the central nervous system to CSF dynamics may be related to frailty. However, neuroimaging studies on frailty focusing specifically on CSF volume rather than the brain parenchyma remain limited. Therefore, in the present study, we analysed cross-sectional data from community-dwelling older adults to examine the association between frailty phenotype, as defined by the Fried criteria [13], and volumes of regions of interest (ROIs), including DESH-related CSF spaces [9] (VS, SF, and SHM) and brain parenchymal regions [14–16] (cerebral and cerebellar cortex). We also exploratorily investigated associations between the ROI volumes and each of the five components of the frailty phenotype [13]—slowness, weakness, low activity, shrinking, and exhaustion. The primary aim of this study was to investigate the relationship between DESH-related CSF space volume and frailty. These findings may contribute to a better understanding of the role of CSF dynamics in frailty and may inform the development of preventive strategies targeting CSF dysfunction.

## Methods

### Study design, setting, and participants

This cross-sectional analysis was conducted using baseline data obtained from a single site (Kumamoto site) of the Japan Prospective Studies Collaboration for Aging and Dementia (JPSC-AD), a cohort study aimed at establishing dementia prevention strategies in Japan, in accordance with the Strengthening the Reporting of Observational studies in Epidemiology guidelines. Data were collected from 1,577 community-dwelling residents aged ≥ 65 years in Arao City, Kumamoto Prefecture, between 2016 and 2017. Details of the survey have been previously reported [17]. Written informed consent was obtained from all participants. In cases where individuals were unable to provide consent owing to dementia, consent was obtained from a family member or legal representative. This study was approved by the Ethics Committee of Kumamoto University (GENOME-333) and was conducted in accordance with the Declaration of Helsinki.

### ROIs

**Fig. 1:**
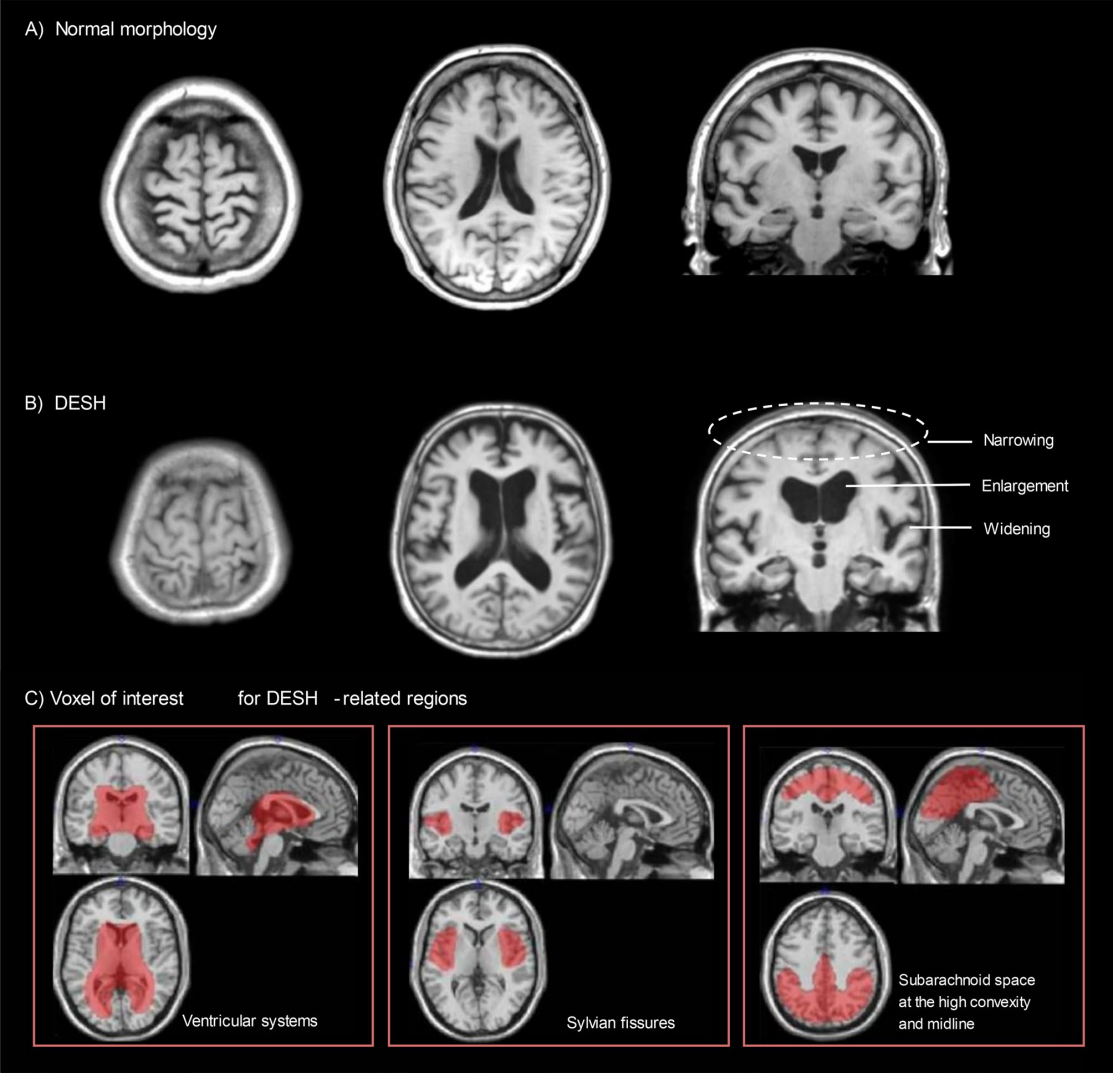
Representative Brain MRI Findings and VOI-based Quantification of CSF in DESH-related Regions. T1-weighted brain MRI images are shown in the axial (left and centre) and coronal (right) planes. **(A)** Normal morphology in a non-frail participant (female, 69 years old; values expressed as percentage of ICV): VS volume = 3.9%, SF volume = 1.4%, SHM volume = 3.5%. **(B)** DESH in a participant with frailty (female, 69 years old), VS volume = 7.1%, SF volume = 1.9%, SHM volume = 1.3%. **(C)** VOI templates illustrating CSF volumes in VS, SF, and SHM, representing DESH-related regions. Abbreviations: DESH, disproportionately enlarged subarachnoid-space hydrocephalus; MRI, magnetic resonance imaging; VOI, volume of interest; ICV, intracranial volume; VS, ventricular system; SF, Sylvian fissures; SHM, subarachnoid space at the high convexity and midline; CSF, cerebrospinal fluid

Quantitative evaluation of DESH-related CSF spaces was performed using an automated brain image segmentation method, as previously described [18, 19]. Briefly, volume of interest (VOI) templates for intracranial volume (ICV), VS, SF, and SHM were created based on a digital phantom Simulated Brain Database aligned with the Montreal Neurological Institute standard space (Fig. 1parameter obtained through grey matter segmentation and anatomical normalisation (SPM8, Wellcome Trust Centre for Neuroimaging, London, UK). The regional volumes (VS, SF, and SHM) were calculated from segmented CSF images. Further methodological details are available in Additional file 1 (Supplemental Methods).

In addition, cerebral and cerebellar cortical volumes were estimated from T1-weighted MRI using FreeSurfer 5.3 (http://surfer.nmr.mgh.harvard.edu/) [20]. Cerebral cortical volume was determined according to the Desikan–Killiany Atlas [21], and cerebellar cortex volume was obtained by summing the volumes of the left and right cerebellar cortices, segmented by FreeSurfer’s automated subcortical segmentation algorithm.

### Imaging

MRI data were acquired using T1-weighted imaging protocols in accordance with the brain MRI guidelines established by the Alzheimer’s Disease Neuroimaging Initiative. Scans were obtained using a Philips Ingenia CX Dual 1.5-Tesla scanner (Philips Healthcare, Best, Netherlands). Imaging was performed with a GE Signa HDxt Ver.23 1.5-Tesla scanner (GE Healthcare, Milwaukee, USA). Further details regarding the MRI acquisition parameters are available in a previous publication [22].

### Frailty assessment

Frailty was diagnosed based on the revised Japanese version of the Cardiovascular Health Study criteria [23]. It comprises five components defined as follows: weakness (grip strength < 28.0 kg for men and < 18.0 kg for women); slowness (comfortable gait speed < 1.0 m/s); low activity (defined as answering “no” to both of the following questions: (1) “Do you engage in moderate levels of physical exercise or sports aimed at health?” and (2) “Do you engage in low levels of physical exercise aimed at health?”); shrinking (defined as answering “yes” to the question “Have you [(unintentionally] lost weight ≥ 2–3 kg in the past 6 months?”); and exhaustion (defined as answering “More than half the days” or “Nearly every day” to the question “[Over the last 2 weeks] Feeling tired or having little energy”). Participants were classified as pre-frail if they met one or two of these components, and as frail if they met three or more.

### Other variables

Apolipoprotein E4 (ApoE4) carrier status was defined by the presence of ε2/ε4, ε3/ε4, or ε4/ε4 alleles. ApoE4 genotype was identified via targeted multiplex polymerase chain reaction sequencing, detecting two single-nucleotide polymorphisms (rs429358 and rs7412). Comorbidity counts included stroke history, injury history, hypertension, diabetes mellitus, obesity, and mild cognitive impairment (MCI). Definitions of these comorbidities were as follows: histories of stroke and injury (including both head and other body areas) were assessed using self-reported questionnaires. Hypertension was defined by systolic/diastolic blood pressure > 140/90 mmHg or current use of antihypertensive medication. Diabetes mellitus was classified according to the 2010 American Diabetes Association criteria [24], defined by fasting glucose ≥ 7.0 mmol/L, random glucose ≥ 11.1 mmol/L, haemoglobin A1c ≥ 6.5%, or ongoing anti-diabetic treatment. Obesity was determined according to criteria from the Japan Society for the Study of Obesity (body mass index ≥ 25 kg/m²) [25]. MCI diagnosis was based on Petersen’s criteria [26], and dementia diagnosis adhered strictly to the Diagnostic and Statistical Manual of Mental Disorders, Third Edition Revised criteria, as detailed in the JPSC-AD protocol [17]. DESH was determined according to the Japanese clinical guidelines endorsed by the Japanese Society of Normal Pressure Hydrocephalus [8] as the coexistence of three features: dilated VS (Evans Index > 0.3), enlarged SF, and tight SHM. This visual rating was performed by one neuroradiologist (N.T.) and two neuropsychiatrists (Y.H. and M.H.), blinded to clinical information.

### Statistical analysis

Descriptive statistics were performed across the three frailty categories (robust, pre-frail, and frail). Continuous variables were compared using the Jonckheere–Terpstra test, and categorical variables using the Cochran–Armitage trend test. In the primary analysis, each of the five ROI volumes was modelled separately with cumulative logit regression (a type of ordinal logistic regression based on the proportional odds assumption). The covariates included age, sex, ApoE4 carrier status (given its reported associations with frailty and brain structure [27, 28]), and comorbidity count [29] (in aggregate form to reduce potential estimation instability). Family-wise error was controlled across the five tests via Benjamini–Hochberg false‐discovery rate (FDR) correction (q < 0.05). Secondary analysis was performed to examine the association between each binary frailty symptom and ROI volume using logistic regression with the same covariates and FDR correction across 25 tests (5 frailty symptoms × 5 ROIs). Sensitivity analysis involved modelling the number of frailty criteria met (0–5) as a simple count score based on previous studies [30, 31] with restricted cubic splines (four knots) using the rms package in R to assess the association with each ROI volume. The same covariates and FDR correction as those used in the primary analysis were applied.

ROI volume distributions showed acceptable skewness (± 1.0) and kurtosis (± 3.0); therefore, no outliers were excluded. ROI volumes were expressed as a percentage of ICV. Missing data were not imputed. All analyses were conducted using IBM SPSS Statistics v29.0 (IBM Corp., Armonk, NY) and R v4.3.3, with two-sided significance set at 5%.

## Results

### Participant selection

**Fig. 2:**
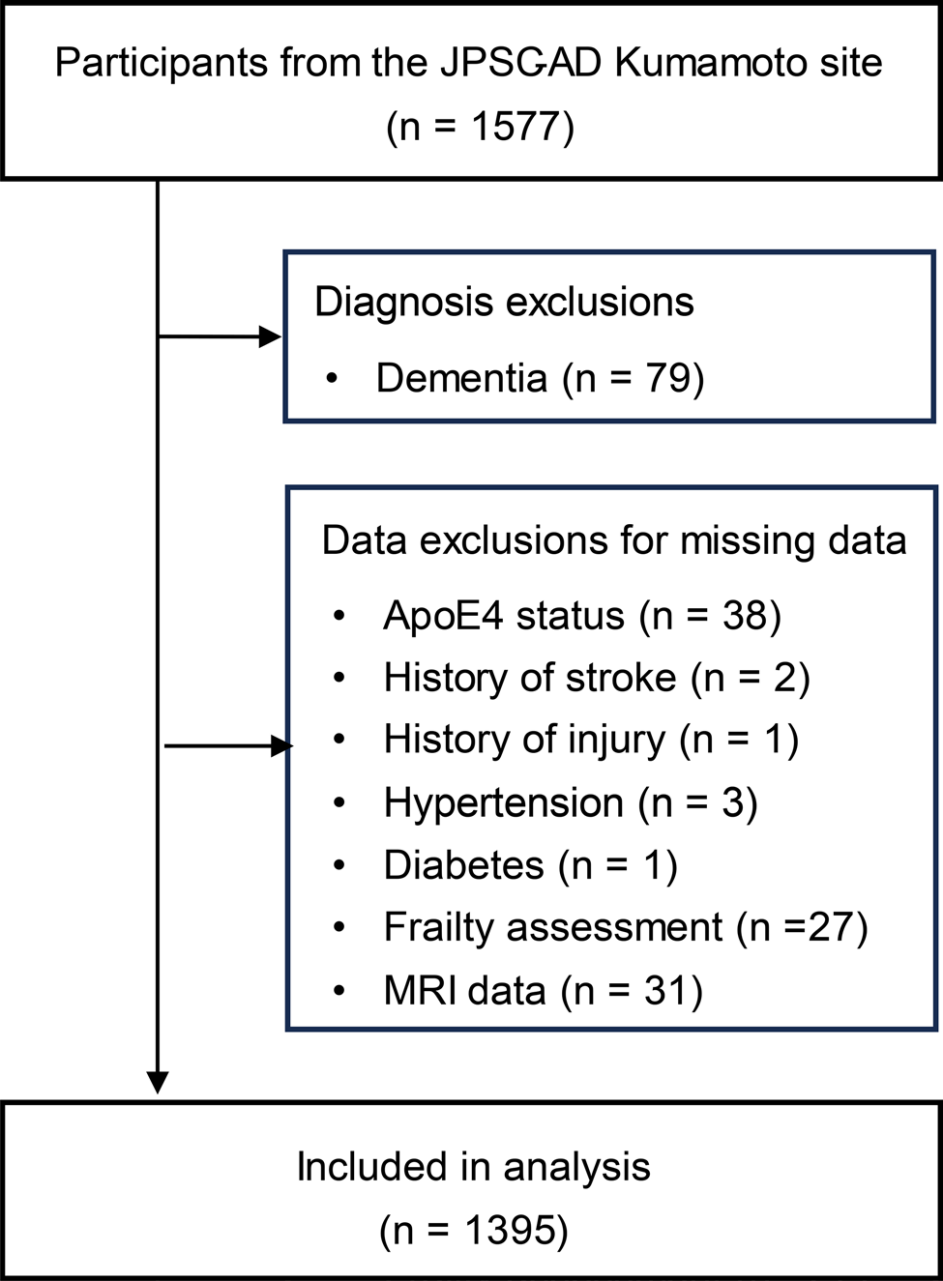
Participant flowchart. Flowchart showing participant selection and exclusion criteria for analysis. The initial sample consisted of 1,577 participants from the JPSC-AD Kumamoto site. Exclusions were made for dementia diagnosis (*n* = 79) and missing data on key variables (ApoE4 status, comorbidities, frailty assessment, and MRI data), resulting in a final analytic sample of 1,395 participants. Abbreviations: JPSC-AD, Japan Prospective Studies Collaboration for Aging and Dementia; ApoE4, apolipoprotein E4; MRI, magnetic resonance imaging

Of the 1,577 community-dwelling older adults who participated in the JPSC-AD study, 79 with dementia and 103 with missing data were excluded, resulting in a final analytic sample of 1,395 participants (Fig. 2).

### Demographic and clinical characteristics

Table 1 presents the participant characteristics according to frailty status: robust (*n* = 843), pre-frail (*n* = 501), and frail (*n* = 51). Trend tests revealed significant associations between frailty status and age (*p* < 0.001), female sex (*p* = 0.013), comorbidity count, and the proportion of participants with stroke history, injury, hypertension, obesity, and MCI.

**Table 1:** Participant characteristics

	Robust	Pre-frailty	Frailty	*p* for trend
	*n* = 843	*n* = 501	*n* = 51	
Age (years)	72.0 (68.0–77.0)	74.0 (69.0–80.0)	81.0 (73.0–86.0)	< 0.001
Women (n)	481 (57.1)	340 (67.9)	37 (72.5)	< 0.001
ApoE4 carrier (n)	136 (16.1)	76 (15.2)	7 (13.7)	0.543
Comorbidity (count)	1.0 (1.0–2.0)	2.0 (1.0–2.0)	2.0 (1.0–3.0)	< 0.001
History of stroke (n)	39 (4.6)	39 (7.8)	6 (11.8)	0.003
History of injury (n)	89 (10.6)	64 (12.8)	11 (21.6)	0.026
Hypertension (n)	595 (70.6)	378 (75.4)	41 (80.4)	0.021
Diabetes mellitus (n)	129 (15.3)	77 (15.4)	13 (25.5)	0.261
Obesity (n)	213 (25.3)	163 (32.5)	14 (27.5)	0.015
Mild cognitive impairment (n)	121 (14.4)	88 (17.6)	24 (47.1)	< 0.001
DESH determination^†^ (n)	11 (1.3)	15 (3.0)	2 (3.9)	0.020
DESH-related region volume				
VS (% of ICV)	3.2 (2.7–3.8)	3.3 (2.8–4.0)	3.8 (3.3–4.4)	< 0.001
SF (% of ICV)	1.3 (1.2–1.5)	1.4 (1.2–1.5)	1.4 (1.3–1.7)	< 0.001
SHM (% of ICV)	3.8 (3.3–4.2)	3.7 (3.2–4.2)	3.5 (2.5–3.9)	0.003

Descriptive statistics are presented for three frailty categories. Continuous variables are expressed as median (interquartile range) and compared using the Jonckheere–Terpstra trend test. Categorical variables are presented as number (percentage) and compared using the Cochran–Armitage trend test

Abbreviations: ApoE4, apolipoprotein E4; DESH, disproportionately enlarged subarachnoid-space hydrocephalus; VS, ventricular system; SF, Sylvian fissures; SHM, subarachnoid space at the high convexity and midline; ICV, intracranial volume

^†^DESH was determined by the coexistence of three features: dilated VS with > 0.3 on the Evans Index, enlarged SF, and tight SHM

### Association between regional ROI volumes and frailty category

**Fig. 3:**
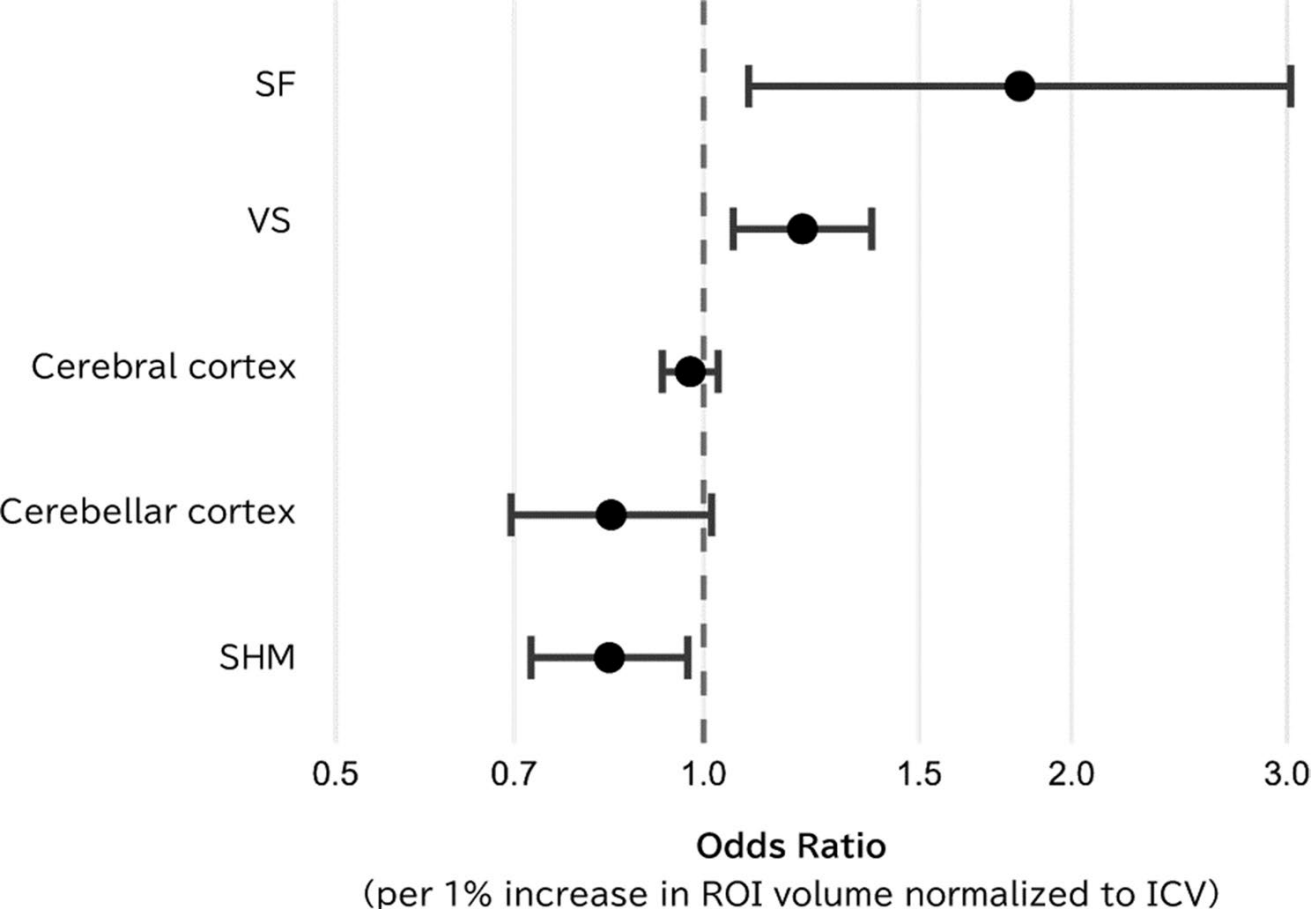
Forest plot of adjusted odds ratios for frailty category. Forest plot showing ORs and 95% CIs per 1% increase in ROI volume from ordinal logistic regression models. Models were adjusted for age, sex, ApoE4 carrier status, and comorbidity count, and FDR correction (Benjamini–Hochberg) was applied across ROIs. The dashed vertical line denotes an OR of 1. Abbreviations: SF, Sylvian fissures; VS, ventricular system; SHM, subarachnoid space at the high convexity and midline; ROI, region of interest; ICV, intracranial volume; OR, odds ratio; CI, confidence interval; ApoE4, apolipoprotein E4; FDR, false-discovery rate

As shown in Fig. 3, ordinal logistic regression with FDR correction indicated that per 1% increase in ROI volume (% of ICV), SF and VS volumes were positively associated with frailty category (SF: odds ratio [OR] 1.81, 95% confidence interval [CI] 1.09–3.02; q = 0.037; VS: OR 1.21, 95% CI 1.06–1.37; q = 0.025), SHM volume was negatively associated (OR 0.84, 95% CI 0.72–0.97; q = 0.037). Cerebral cortex and cerebellar cortex volumes showed no significant associations (both q > 0.05). Full regression results are provided in Additional file 1: Table S1.

### Association between five frailty components and ROI volume

**Fig. 4:**
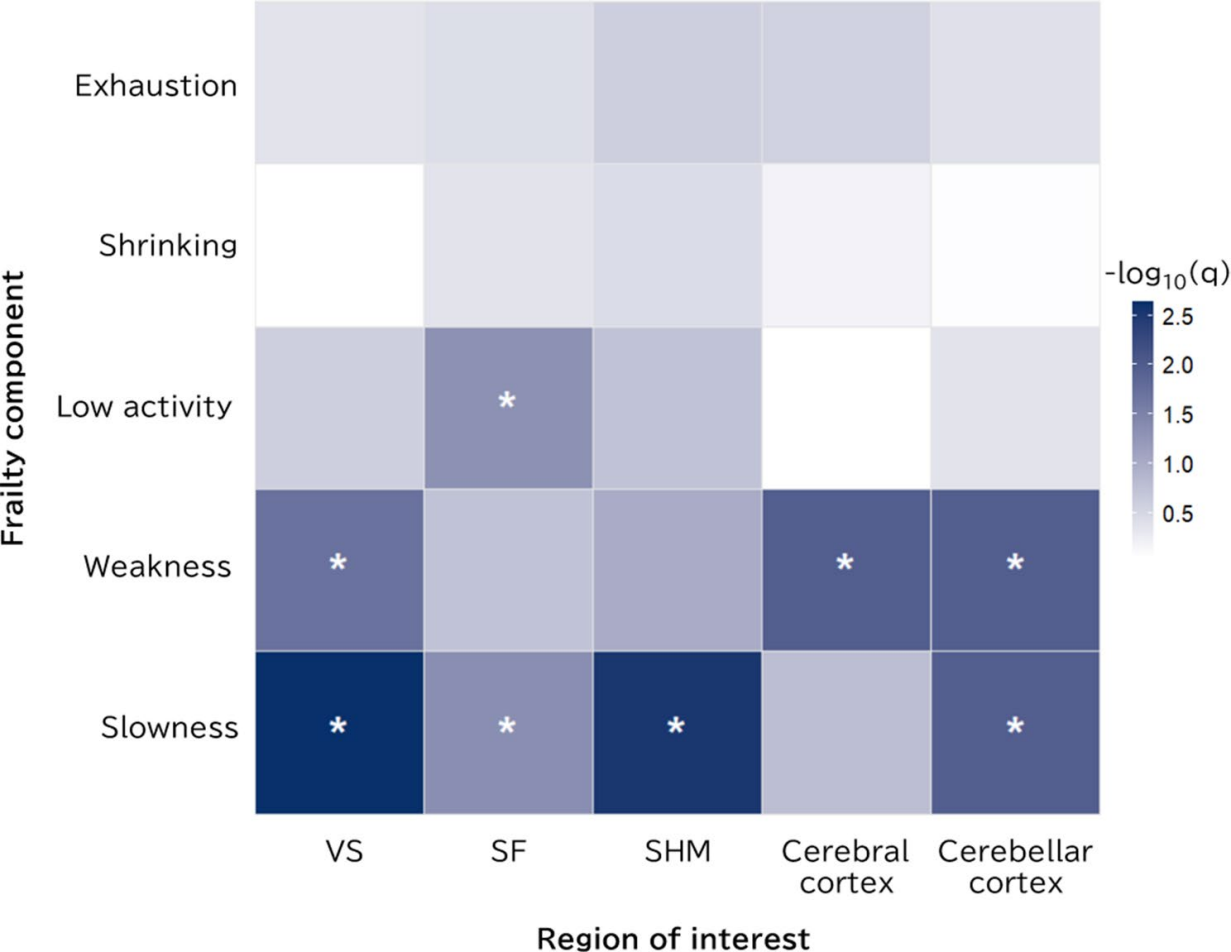
Heatmap of associations between five frailty components and ROI volumes. The heatmap shows − log₁₀(q-values) from logistic regression models evaluating the association between each frailty component and ROI volume (per 1% increase, normalised to ICV), with FDR correction for multiple comparisons. Asterisks indicate statistical significance (q < 0.05). Abbreviations: VS, ventricular system; SF, Sylvian fissures; SHM, subarachnoid space at the high convexity and midline; ROI, region of interest; ICV, intracranial volume; FDR, false-discovery rate

Logistic regression analysis with FDR correction was performed to evaluate associations between five frailty components—slowness, weakness, low activity, shrinking, and exhaustion—and ROI volume (per 1% increase, expressed as % of ICV). As shown in Fig. 4, slowness was significantly associated with greater VS (β = 0.40, q = 0.002) and SF (β = 1.01, q = 0.045) volumes and lower SHM (β = − 0.44, q = 0.003) and cerebellar cortex (β = − 0.51, q = 0.010) volumes. Weakness was significantly associated with greater VS volume (β = 0.27, q = 0.019) and lower cerebral cortex (β = − 0.12, q = 0.010) and cerebellar cortex (β = − 0.45, q = 0.010) volumes. Low activity was associated with greater SF volume (β = 0.80, q = 0.048). No significant associations were observed between shrinking or exhaustion and any ROI after FDR correction. Full statistical results for all frailty components and ROIs are provided in Additional file 1: Table S2.

### Sensitivity analysis: associations between ROI volumes and frailty component count

**Fig. 5:**
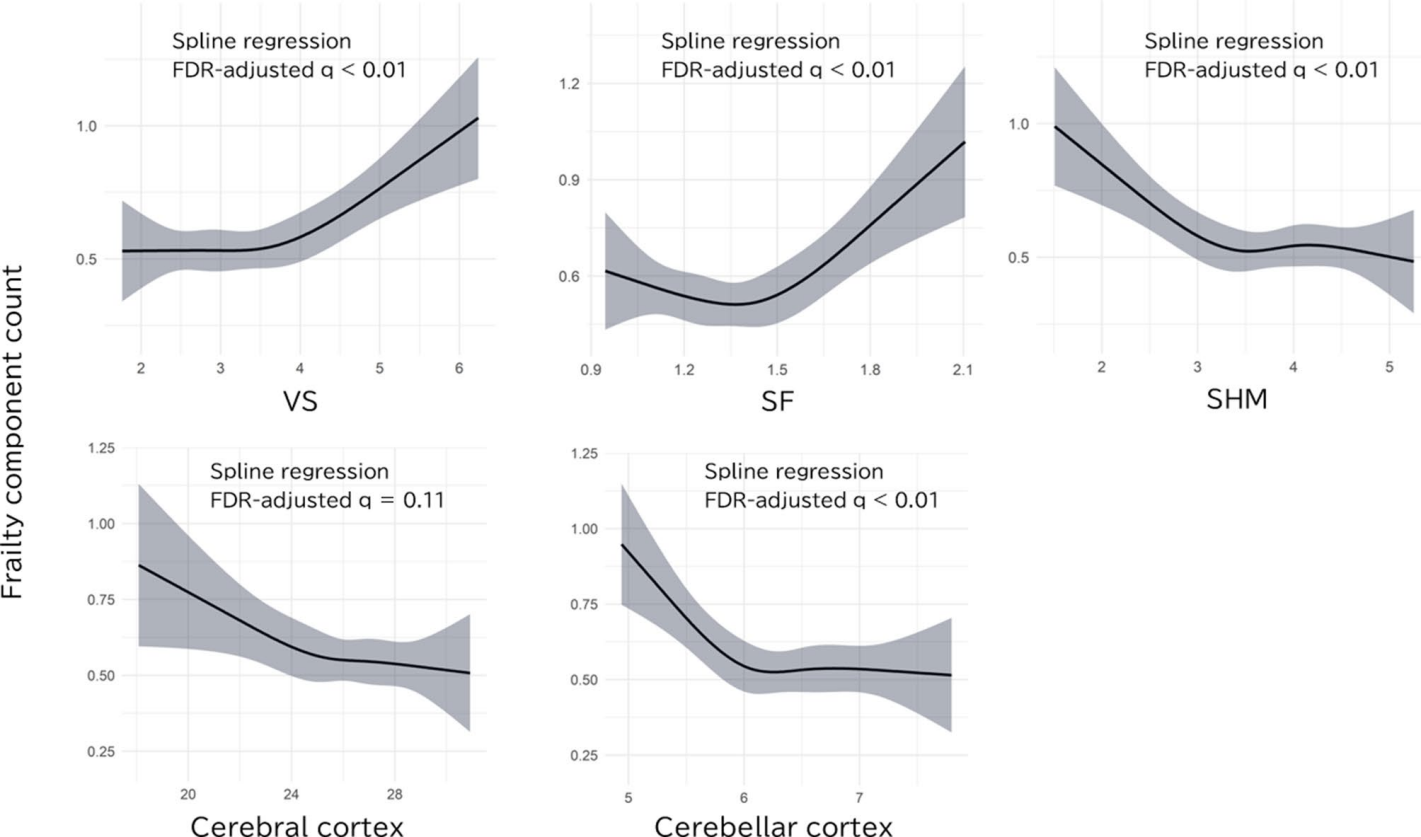
Spline curves for the association between ROI volume and frailty component count. Predicted frailty component count based on the Fried phenotype is plotted against each ROI volume using restricted cubic spline regression. Shaded areas represent 95% confidence intervals. FDR correction was applied. Abbreviations: FDR, false-discovery rate; VS, ventricular system; SF, Sylvian fissures; SHM, subarachnoid space at the high convexity and midline; ROI, region of interest

Restricted cubic spline regression was performed to assess associations between each ROI volume and frailty component count based on the Fried phenotype. As shown in Fig. 5, significant associations were observed for the VS (q = 0.001), SF (q = 0.001), and SHM (q = 0.001), with greater ventricular and SF volumes and lower SHM volume being associated with increased frailty component count. No significant association was observed for cerebral cortex volume (q = 0.107). The direction of these associations was consistent with that observed for frailty categories. See Additional file 1: Table S3 for the detailed results.

## Discussion

We found that DESH-related CSF spaces are associated with frailty in community-dwelling older adults. The volumes of three DESH-related regions—VS, SF, and SHM—were significantly associated with frailty, and all three regions were related to the slowness phenotype. In contrast to previous studies that predominantly focused on the brain parenchyma in relation to frailty, the present study focused on CSF space volumes—reflecting impaired CSF dynamics—revealing that they may also be involved as a neural factor contributing to the expression of frailty phenotypes.

The observed association between DESH-related region volume and frailty is consistent with previous reports. In prior genome-wide association studies, *MLLT10* was identified as a genetic locus associated with iNPH [32] and, in a separate study, as associated with frailty [33]. Taken together, these findings raise the possibility that DESH-related brain changes and frailty may at least partly be influenced by common genetic factors. Moreover, population-based studies [10, 34–36] indicate that both DESH findings and frailty are associated with ageing, neurodegenerative disorders (e.g., Alzheimer’s disease), and cerebrovascular diseases. In a clinical study [37] of individuals suspected of having iNPH based on clinical symptoms and imaging findings (including Evans Index > 0.3), Alzheimer’s pathology was observed in 72% and cerebrovascular diseases in 67%. The mean frailty index in that population was 0.32, corresponding to a range from pre-frailty to frailty according to Fried’s criteria [38]. These findings, taken together with our community-based findings, suggest that frailty in some older adults may reflect underlying iNPH-related processes.

Among frailty components, slowness was found to be significantly associated with volumes of VS, SF, SHM, and the cerebellar cortex. Several studies [39–41] suggest that volumes of ventricles and cerebellar cortex are associated with gait ability in older adults. Although the precise mechanism underlying the relationship between DESH and gait disturbance is unknown, this may be due to compression of the corticospinal tract, which controls lower limb movements, via enlarged VS and SHM [42]. No studies have directly assessed the relationships between SF volume and low activity or between VS volume and weakness. However, apathy is one of the most prevalent neuropsychiatric manifestations of iNPH [43], and CSF drainage has been associated with improvements in grip strength [44] and lower extremity muscle strength [45]. These findings support those of the present study. In the present study, the objectively assessed frailty components slowness and weakness showed more widespread associations with DESH-related ROI volumes than the subjective components such as low activity, exhaustion, and weight loss, for which only low activity was associated with SF after correction for multiple comparisons. Subjective components are more susceptible to psychological, cultural, and reporting biases and may therefore be less sensitive to underlying structural brain changes. Indeed, previous research has shown that subjective assessments of frailty-related performance do not necessarily correspond well to objective assessments [46], indicating that associations between DESH-related CSF volumes and subjectively assessed components should be interpreted with caution and ideally replicated in independent cohorts.

The relationship between DESH-related CSF spaces and frailty may offer insights into the clinical implications of frailty in the context of iNPH. For example, interventions targeting lifestyle-related and vascular risk factors—such as hypertension, obesity, physical inactivity, and vascular disease—as well as chronic inflammation [47, 48] may also serve as effective preventive strategies against frailty. In addition, CSF drainage and rehabilitation after iNPH onset have been suggested to improve gait, balance, and activities of daily living [49–51]. These findings may provide useful information for personalised interventions for frailty using DESH findings.

A strength of this study is its analysis of the relationship between DESH-related CSF volumes and frailty in approximately 1,400 community-dwelling older adults, using quantitative measurements of CSF and brain parenchymal volumes. To our knowledge, only a few studies have been conducted to directly quantify CSF volumes in DESH-related regions in such a large population. However, this study has some limitations. First, owing to its cross-sectional design, a causal relationship could not be established between DESH-like brain morphological changes and frailty. Second, the number of participants with frailty was small, which may have reduced the statistical power. Third, although we excluded individuals with dementia and adjusted for overall comorbidity burden, we did not systematically exclude those with non-neurological conditions, such as orthopaedic, internal medical, or malignant diseases. Therefore, residual confounding due to these conditions and factors, such as MRI scanner type, cannot be entirely ruled out. In the additional sensitivity analysis (Additional file 1: Table S4), which was further adjusted for individual comorbidities, scanner type, and pain status, the direction of associations of the volumes of the VS, SF, and SHM with frailty was confirmed to be consistent. Fourth, in this study, frailty was defined solely according to the Fried phenotype; therefore, our findings may not generalise to other operational definitions, such as deficit-accumulation frailty indices.

## Conclusion

This study revealed that the volumes of DESH-related regions—the VS, SF, and SHM—are significantly associated with frailty diagnosis. Assessment of DESH-related CSF spaces may aid personalised frailty interventions, and preserving normal CSF dynamics could be key to frailty prevention. Prospective investigations are required to clarify the causal relationship between CSF dynamics-related DESH findings and frailty risk.

## Supplementary Information

Below is the link to the electronic supplementary material.

Supplementary Material 1

## Data Availability

No datasets were generated or analysed during the current study.

## Abbreviations

ApoE4: Apolipoprotein E4
CI: Confidence interval
CSF: Cerebrospinal fluid
DESH: Disproportionately enlarged subarachnoid-space hydrocephalus
FDR: False-discovery rate
ICV: Intracranial volume
iNPH: Idiopathic normal pressure hydrocephalus
JPSC-AD: Japan Prospective Studies Collaboration for Aging and Dementia
MCI: Mild cognitive impairment
MRI: Magnetic resonance imaging
OR: Odds ratio
ROI: Region of interest
SF: Sylvian fissures
SHM: Subarachnoid space at the high convexity and midline
VOI: Volume of interest
VS: Ventricular system
