# Supplementary material for: Association between cerebrospinal fluid volume and frailty in community-dwelling older adults: a cross-sectional study
Source: Fluids Barriers CNS. 2026 Jan 24;23:36. doi: 10.1186/s12987-026-00761-1 (PMC12930604; doi:10.1186/s12987-026-00761-1)
Supplement: Supplementary file 1 — Supplementary Material 1 [file 12987_2026_761_MOESM1_ESM.pdf]

Association Between Cerebrospinal Fluid Volume and Frailty in Community-dwelling Older Adults: A Cross-Sectional Study

Additional file 1

Supplemental Methods and Results

Contents

- Supplemental Methods ……………………………………………………………………2

- Table S1……………………………………………………..……………………………4

- Table S2…………………..………………………………………………………………5

- Table S3……………………………………………………..……………………………7

- Table S4……………………………………………………..……………………………8

Supplemental Methods

Image Processing and VOI-Based Quantification

To quantitatively assess disproportionately enlarged subarachnoid space hydrocephalus (DESH)-related regions, we utilized an automatic volumetric segmented brain imaging system that was modified to evaluate idiopathic normal-pressure hydrocephalus[1 2]. We prepared voxel of interest (VOI) templates for intracranial volume, ventricular system (VS), Sylvian fissure (SF), and subarachnoid space at the high convexity and midline (SHM) (figure 1), as previously described. [5] Each regional VOI template was generated using a digital phantom of the Simulated Brain Database (https://www.bic.mni.mcgill.ca/brainweb/) according to the standard Montreal Neurological Institute space with the contours of each structure manually delineated. The SHM VOI template was manually produced based on the results of a previous study on voxel-based morphometry in patients and normal controls. [6] For this process, the MRI of each participant was segmented into the gray matter (GM), WM, and CSF using the SPM8 segmentation program (https://www.fil.ion.ucl.ac.uk/spm/software/spm8/). The GM template derived from the Simulated Brain Database was spatially transformed into a GM image for each participant, and a normalization parameter was determined using SPM8 and the Diffeomorphic Anatomical Registration Through Exponentiated Lie Algebra technique. Using this normalization parameter, which functions like the reverse parameter generated during anatomical normalization to a standard brain, the intracranial volume and VS, SF, and SHM VOI templates were transformed into each participant's space. The intracranial volume was adjusted using images derived from segmented GM, WM, and CSF images. Segmented GM (WM) images were derived by calculating GM (WM) areas with voxels from the intracranial volume VOI template. The CSF volumes of the VS, SF, and

SHM were calculated using individual transformed VS, SF, and SHM subarachnoid space VOI templates. Each regional volume was normalized to the total intracranial volume.

References

1. Ishii K, Soma T, Kono AK, et al. Automatic volumetric measurement of segmented brain structures on magnetic resonance imaging. *Radiat Med* 2006;24(6):422-30. doi: 10.1007/s11604-006-0048-8

2. Ishii K, Soma T, Shimada K, et al. Automatic volumetry of the cerebrospinal fluid space in idiopathic normal pressure hydrocephalus. *Dement Geriatr Cogn Dis Extra* 2013;3(1):489-96. doi: 10.1159/000357329 [published Online First: 20131220]

Supplemental Results

Table S1. Associations between ROI volumes and frailty category based on ordinal logistic regression

Frailty category	β	SE	Wald χ^2	p	q (FDR)	OR [95%CI]
VS	0.19	0.07	7.86	0.005	0.026	1.21 [1.06 –1.37]
SF	0.60	0.26	5.24	0.022	0.037	1.81 [1.09 –3.02]
SHM	-0.18	0.08	5.57	0.018	0.037	0.84 [0.72 –0.97]
Cerebral Cortex	-0.02	0.03	0.86	0.354	0.356	0.98 [0.93 –1.03]
Cerebellar cortex	-0.17	0.10	3.26	0.071	0.089	0.84 [0.70 –1.01]

ROI volumes were entered as explanatory variables. Regression coefficients (β), standard errors (SE), Wald χ^2 statistics (all tests with 1 degree of freedom), unadjusted *p*-values, false discovery rate–adjusted *q*-values (FDR), and odds ratios (OR) with 95% confidence intervals (CIs) are reported.

Abbreviations: ROI, region of interest; VS, ventricle systems; SF, Sylvian fissures; SHM, subarachnoid space at the high convexity and midline; FDR, false discovery rate.

Table S2. Summary of regression results for associations between five frailty components and ROI volumes

	β	SE	Wald χ^2	p	q (FDR)	OR [95%CI]
**Slowness**						
VS	0.40	0.10	15.26	<0.001	0.002	1.50 [1.22–1.83]
SF	1.01	0.40	6.24	0.012	0.045	2.74 [2.24–6.04]
SHM	-0.44	0.12	13.52	<0.001	0.003	0.64 [0.51–0.81]
Cerebral Cortex	-0.08	0.04	3.42	0.064	0.161	0.93 [0.85–1.00]
Cerebellar cortex	-0.51	0.17	9.47	0.002	0.010	0.60 [0.43–0.83]
**Weakness**						
VS	0.27	0.09	8.04	0.005	0.019	1.31 [1.09–1.57]
SF	0.63	0.37	2.89	0.089	0.186	1.88 [0.91–3.88]
SHM	-0.22	0.11	4.42	0.036	0.099	0.80 [0.65–0.99]
Cerebral Cortex	-0.12	0.04	9.61	0.002	0.010	0.89 [0.83–0.96]
Cerebellar cortex	-0.45	0.15	9.48	0.002	0.010	0.64 [0.48–0.85]
**Low activity**						
VS	0.12	0.08	2.13	0.144	0.258	1.13 [0.96–1.33]
SF	0.80	0.33	5.87	0.015	0.048	2.24 [1.17–4.29]
SHM	-0.17	0.10	2.99	0.084	0.186	0.85 [0.70–1.02]
Cerebral Cortex	0.01	0.04	0.03	0.870	0.884	1.01 [0.94–1.08]
Cerebellar cortex	-0.12	0.13	0.84	0.359	0.427	0.89 [0.70–1.14]

**Shrinking**						
VS	0.01	0.10	0.02	0.884	0.884	1.01 [0.84–1.23]
SF	-0.36	0.39	0.85	0.356	0.427	0.69 [0.32–1.51]
SHM	0.13	0.11	1.43	0.232	0.362	1.14 [0.92–1.43]
Cerebral Cortex	0.02	0.04	0.34	0.562	0.639	1.02 [0.95–1.11]
Cerebellar cortex	0.04	0.14	0.08	0.782	0.849	1.04 [0.79–1.37]
**Exhaustion**						
VS	0.12	0.12	0.95	0.329	0.427	1.13 [0.89–1.43]
SF	0.53	0.47	1.27	0.259	0.381	1.70 [0.68–4.30]
SHM	-0.20	0.14	2.18	0.140	0.258	0.82 [0.62–1.07]
Cerebral Cortex	-0.07	0.05	1.96	0.162	0.270	0.93 [0.85–1.03]
Cerebellar cortex	-0.20	0.19	1.14	0.285	0.396	0.82 [0.57–1.18]

Regression coefficients (β), standard errors (SE), Wald χ^2 statistics (all tests with 1 degree of freedom), unadjusted *p*-values, false discovery rate–adjusted *q*-values (FDR), and odds ratios (OR) with 95% confidence intervals (CIs) are reported.

Abbreviations: ROI, region of interest; VS, ventricle systems; SF, Sylvian fissures; SHM, subarachnoid space at the high convexity and midline; FDR, false discovery rate.

Table S3. Spline regression results for associations between ROI volumes and frailty component count

Frailty component count	Spline p	Spline q (FDR)	Nonlinear p	Nonlinear q (FDR)
VS	<0.001	0.001	0.051	0.064
SF	<0.001	0.001	0.004	0.022
SHM	<0.001	0.001	0.029	0.048
Cerebral Cortex	0.107	0.107	0.478	0.478
Cerebellar cortex	<0.001	0.001	0.013	0.032

Associations between ROI volumes and frailty component count were examined using restricted cubic spline regression (4 knots), adjusted for age, comorbidities, gender, and ApoE4 status.

p-values were obtained from Wald tests; q-values represent false discovery rate–adjusted p-values (Benjamini–Hochberg method).

Abbreviations: ROI, region of interest; VS, ventricle systems; SF, Sylvian fissures; SHM, subarachnoid space at the high convexity and midline; FDR, false discovery rate; ApoE4, apolipoprotein E4.

Table S4. Spline regression results for associations between ROI volumes and frailty component count with additional covariate adjustment

Frailty component count	Spline p	Spline q (FDR)	Nonlinear p	Nonlinear q (FDR)
VS	0.001	0.002	0.069	0.086
SF	<0.001	0.002	0.004	0.021
SHM	0.005	0.005	0.051	0.085
Cerebral Cortex	0.002	0.002	0.481	0.481
Cerebellar cortex	0.002	0.002	0.033	0.082

Associations between ROI volumes and frailty component count were examined using restricted cubic spline regression (4 knots), adjusted for age, gender, education (<10 years), ApoE4 status, MRI scanner type, history of stroke, history of injury, hypertension, diabetes mellitus, obesity, mild cognitive impairment, and presence of pain.

p-values were obtained from Wald tests; q-values represent false discovery rate–adjusted p-values (Benjamini–Hochberg method).

Abbreviations: ROI, region of interest; VS, ventricle systems; SF, Sylvian fissures; SHM, subarachnoid space at the high convexity and midline; FDR, false discovery rate; ApoE4, apolipoprotein E4.
